# Identification of Lynch Syndrome in Patients with Endometrial Cancer Based on a Germline Next Generation Sequencing Multigene Panel Test

**DOI:** 10.3390/cancers14143406

**Published:** 2022-07-13

**Authors:** Yoo-Na Kim, Min Kyu Kim, Young Joo Lee, Youngeun Lee, Ji Yeon Sohn, Jung-Yun Lee, Min Chul Choi, Migang Kim, Sang Geun Jung, Won Duk Joo, Chan Lee

**Affiliations:** 1 Department of Obstetrics and Gynecology, Institute of Women’s Life Medical Science, Yonsei University College of Medicine, Seoul 03722, Korea; heartonbrainmd@yuhs.ac (Y.-N.K.); yjlee1213@yuhs.ac (Y.J.L.); 2 Division of Gynecologic Oncology, Department of Obstetrics & Gynecology, Samsung Changwon Hospital, Sungkyunkwan University of Medicine, Changwon 51353, Korea; minkyukim@skku.edu (M.K.K.); duddms021@gmail.com (Y.L.); 3 Department of Laboratory Medicine, Eone Laboratories, Incheon 22014, Korea; jysohn@eonelab.co.kr; 4 Comprehensive Gynecologic Cancer Center, CHA Bundang Medical Center, CHA University, Seongnam 13496, Korea; mgkim87@chamc.co.kr (M.K.); sgoncol@chamc.co.kr (S.G.J.); wdjoo@chamc.co.kr (W.D.J.); chanoncology@chamc.co.kr (C.L.)

**Keywords:** endometrial cancer, Lynch Syndrome, next generation sequencing, prevalence

## Abstract

**Simple Summary:**

Lynch Syndrome (LS) is a hereditary cancer syndrome caused by an autosomal dominant mutation in one of the DNA mismatch repair (MMR) genes. Understanding the clinical traits of endometrial cancer, which is one of the most frequent as well as the earliest diagnosed cancer types of LS-associated cancers, has the potential to offer tailored screening programs and lifesaving interventions. Whereas most large-scale studies of LS-associated endometrial cancers are based on cohorts in Western countries, studies from a predominantly Korean population are relatively few. We conducted a multi-center, retrospective study of patients with endometrial cancer who underwent germline multigene panel testing that included MMR genes. Based on the analysis of 204 patients with endometrial cancer who underwent germline multigene panel testing, we investigated the prevalence of LS and non-LS mutation and the relative contribution of each MMR gene mutation that, in composite, led to the clinical phenotype of LS-associated endometrial cancer patients in a predominantly Korean population.

**Abstract:**

We aimed to investigate the prevalence and relative contributions of LS and non-LS mutations in patients with endometrial cancer in Korea. We retrospectively reviewed the medical records of 204 patients diagnosed with endometrial cancer who underwent a germline next generation sequencing multigene panel test covering *MLH1*, *MSH2*, *MSH6*, *PMS2*, and *EPCAM* at three tertiary centers. Thirty patients (14.7%) with pathogenic mutations (12 *MLH1*; 6 *MSH2*; 10 *MSH6*; 2 *PMS2*) and 20 patients (9.8%) with 22 unclassified variants (8 *MLH1*; 8 *MSH2*; 2 *MSH6*; 3 *PMS2*; 1 *EPCAM*) were identified. After excluding four close relatives of a proband, the prevalence of LS was 13.0% (26/200). Patients with LS were more likely than those with sporadic cancer to be younger at diagnosis (48 vs. 53 years, *p* = 0.045) and meet the Amsterdam II criteria (66.7 vs. 3.5%, *p* < 0.001). Non-endometrioid histology was more prevalent in patients with *MSH6* or *PMS2* mutations (41.7%) than those with *MLH1* or *MSH2* mutations (5.6%, *p* = 0.026). In this pre-selected cohort of endometrial cancer patients who underwent next generation sequencing, the prevalence of LS was 13%, thus supporting the use of gene panel testing for endometrial cancer patients.

## 1. Introduction

Lynch Syndrome (LS) is a hereditary cancer syndrome caused by an autosomal dominant mutation in one of the DNA mismatch repair (MMR) genes, including *MLH1, MSH2, MSH6*, and *PMS2* [1]. Deletions in the *EPCAM* gene, which lead to hypermethylation of the *MSH2* promoter and, consequently, *MSH2* silencing, also cause LS [2]. Individuals with LS have an increased risk of colon cancer; synchronous/metachronous extra-colonic LS-associated cancers involving the endometrium, ovaries, stomach, pancreatobiliary tract, urinary tract, small bowel, and brain (usually glioblastomas); sebaceous adenomas; sebaceous carcinomas; and keratoacanthomas. Clinical manifestations of LS vary widely according to the specific types of LS-associated cancers. For instance, patients may present with bloody stool in the case of colon cancer and vaginal bleeding in endometrial cancer, yet the cancer-associated symptom may not be apparent in cases of early-stage cancer, especially in certain types of cancers such as cancers of the ovary or pancreatobiliary tract. Rather than clinical symptoms, LS is characterized by an early age of onset, more than one primary cancer within an individual, and a family history of multiple LS-associated cancers. Early screening of patients at risk of LS has the potential to offer tailored screening programs and lifesaving interventions for patients and their family members [3,4,5,6]. Furthermore, endometrial cancer is likely to be the first cancer diagnosis for approximately half of patients with LS, preceding colon cancer by an average of 11 years. This underscores the importance of understanding the clinical traits of LS-associated endometrial cancer [7].

The lifetime risk of endometrial cancer in patients with LS is reported to be 40–60%, based on studies conducted predominantly in Western countries [8,9]. Although alterations in any one of the MMR genes may result in microsatellite instability and result in LS, each gene has slightly different clinical and histopathological manifestations [10,11]. For example, patients with *MLH1* mutations are frequently diagnosed at a younger age, and patients with *MSH6* mutations are more likely to develop gynecological cancer than colorectal cancer. A further contribution to the heterogeneity among patients with LS is that the relative prevalence and type of mutation varies according to the ethnic background and the study setting [12,13,14].

The current guidelines for determining who should undergo genetic testing for LS are known as “universal screening” guidelines. They recommend screening endometrial and colon tumors for defective DNA MMR using immunohistochemistry (IHC) and/or a microsatellite instability (MSI) test and making testing decisions based on personal/family cancer history criteria, such as the Amsterdam II criteria. However, not all centers have adopted “universal tumor screening”, and Amsterdam II criteria are very stringent, such that they miss as many as 68% of patients with LS [15]. With respect to the MSI test, at the current level of development, there are challenges with utilizing the MSI test due to biological and technical heterogeneity [16]. Meanwhile, the advent of next generation sequencing (NGS) tests, which allow the cost-effective analysis of multiple genes simultaneously, and the resulting development of multigene panels have allowed low-cost genetic testing of individuals with a risk of developing hereditary cancers. An earlier study reported that various gene mutations, including those in MMR genes, were identified in 14.7% of patients with endometrial cancer using a multigene panel test [17].

Most large-scale studies of LS-associated endometrial cancers are based on cohorts in Western countries, such as the US, Canada, or European countries [8,9,11]. However, studies of LS-associated endometrial cancers from a predominantly Korean population are relatively few [6,18,19,20,21]. Therefore, we conducted a multi-center, retrospective study of patients with endometrial cancer who underwent germline multigene panel testing that included MMR genes. The goal of this study was to investigate the prevalence of LS and non-LS mutations and the relative contribution of each MMR gene mutation that, in composite, led to the clinical phenotype of LS-associated endometrial cancer patients in a predominantly Korean population.

## 2. Materials and Methods

### 2.1. Patients and Clinical Variables

All patients who were pathologically diagnosed with endometrial cancer and underwent multigene panel-based germline genetic testing according to the institutional protocol for (i) a family history of LS-associated cancers, (ii) abnormal tumor test results, or (iii) endometrial cancer histology were identified at three institutions from January 2015 to December 2021. All patients who were pathologically diagnosed with endometrial cancer and underwent multigene panel-based germline genetic testing were identified at three institutions from January 2015 to December 2021. The decision was to undergo germline next generation sequencing-based testing on (i) a family history of LS-associated cancers, (ii) abnormal tumor test results, or (iii) endometrial cancer histology. However, due to the lack of pre-specified, shared protocols among institutions, the decision to perform germline next generation sequencing was based on individual clinicians. Institutional review board approval was obtained from all study sites (CHAMC 2021-04-047, YUHS 4-2022-0247, SCMC 2021-07-005).

Patient charts were reviewed for the age of endometrial cancer diagnosis, a personal history of cancer, and pedigree with age at diagnosis of LS-associated cancers, to assess the Amsterdam II criteria. Amsterdam II criteria were defined as follows: (i) the family included three or more relatives with an LS-associated cancer, (ii) one affected patient was a first-degree relative of the other two patients, (iii) at least two successive generations were affected, (iv) cancer was diagnosed before the age of 50 years in at least one affected relative, and (v) familial adenomatous polyposis was excluded for any cases of colorectal cancer [22]. The basic profile, such as the stage and histological subtype, were obtained for endometrial cancer diagnoses. Based on pre-operative magnetic resonance images and pathological reports, the primary tumor sites were classified as the uterine corpus or lower uterine segment. The IHC staining results of the four MMR proteins and MSI test results were collected if available.

### 2.2. MultiGene Next Generation Sequencing Test

Germline testing was performed using at least one of the following customized, targeted capture sequencing panels: OncoRisk (Celemics, Seoul, Korea), RiskCare Cancer Panel (Eone laboratory, Incheon, Korea), or Hereditary Cancer Syndrome Panel 21 (GCgenome, Yongin, Korea). These panels cover all coding sequences and intron-exon boundaries of the coding exons of known cancer predisposition genes. These tests were performed at commercial clinical laboratories certified by the Korean Institute of Genetic Testing Evaluation. In this study, the test results of 22 genes (including *MLH1*, *MSH2*, *MSH6*, *PMS2*, and *EPCAM*) were extracted from the gene panel test results and analyzed. The full gene list is presented in Table S1. Based on the guidelines of the American College of Medical Genetics and Genomics 2017, genomic variants were evaluated and classified as either pathogenic, likely pathogenic, or variants of uncertain significance (VUS). Pathogenic or likely pathogenic germline variants were defined as pathogenic mutations.

### 2.3. Statistical Analysis

Continuous variables were compared using a Student’s *t*-test or Mann-Whitney test, and categorical variables were compared using a chi-square test or Fisher’s exact test, as appropriate. Predictive performance for a given test modality was assessed with respect to the identification of germline LS as the reference. A *p* < 0.05 was considered to be statistically significant, and all analyses and figure generation were performed using R version 3.6.1 (R Foundation for Statistical Computing, Vienna, Austria).

## 3. Results

**Figure 1:**
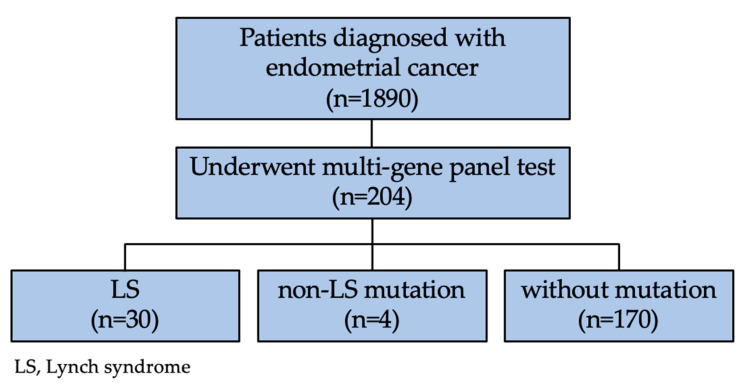
Patient flow chart.

A total of 204 endometrial cancer patients who underwent multigene panel testing at three institutions were identified (Figure 1). The baseline characteristics of the patients are summarized in Table 1. The median age at diagnosis was 52 years (range 23–84). The majority of patients had uterine-confined early-stage cancer, with predominantly an endometrioid histology. The tumor site was the uterine corpus in 84.3% of patients and the lower uterine segment in 15.7% of patients. A personal history of LS-associated cancer, other than endometrial cancer, was found in 41.2% (84/204) of patients. Based on the time of diagnosis, synchronous diagnosis of LS-associated cancer was found in 41 patients (20.1%), with concurrent endometrial and ovarian cancer diagnosis occurring in approximately two-thirds of these patients. The most frequently observed metachronous diagnosis was ovarian cancer (19.1%), followed by breast (11.3%) and colon (10.8%) cancer (data not provided). Overall, 55.9% of patients had a family history of LS-associated cancers, and 12.7% met the Amsterdam II criteria. Among the patients who underwent IHC and MSI testing, the rates of MMR deficiency and high MSI were 42.9% (57/133) and 22.9% (22/96), respectively.

**Figure 2:**
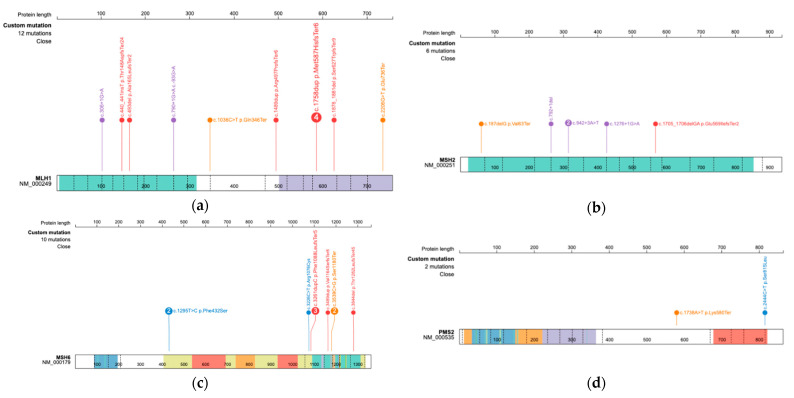
The location and type of (**a**) *MLH1*, (**b**) *MSH2*, (**c**) *MSH6*, and (**d**) *PMS2* mutations within each chromosome are described with a mutation map.

Thirty patients (14.7%) were found to have pathogenic or likely pathogenic variants in DNA MMR genes, including *MLH1* (n = 12), *MSH2* (n = 6), *MSH6* (n = 10), and *PMS2* (n = 2). After excluding four close relatives (sisters or daughters) who were part of a proband’s family, the prevalence of LS was 13.0% (26/200) in this cohort. The detailed clinical information and genomic mutation profile of the LS patients with pathogenic mutations are presented in Table 2, and the location and type of mutation within each chromosome are presented as a mutation map in Figure 2. Comparing patients with LS to those with sporadic endometrial cancer (Table 3), patients with LS were younger (median age, 48 vs. 53 years; *p* = 0.045) and more likely to have a family history of LS-associated cancer (86.7% vs. 49.4%, *p* = 0.001). Patients with LS were more likely than those with sporadic endometrial cancer to meet the Amsterdam II criteria (66.7% vs. 3.5%, *p* = 0.001).

Clinical comparisons between high-penetrance (*MLH1* or *MSH2*) and low-penetrance genes (*MSH6* or *PMS2*) are summarized in Table 4. Non-endometrioid histology was more frequently observed in patients with *MSH6* or *PMS2* mutations compared to those with *MLH1* or *MSH2* mutations (41.7% vs. 5.6%, *p* = 0.026). The non-endometrioid histology cancer types found in patients with *MSH6* mutations included serous (n = 1), neuroendocrine (n = 1), dedifferentiated (n = 1), and mixed type (n = 1) cancers, whereas only one case of mixed cell type cancer was found among patients with *PMS2* mutations.

Twenty-two VUS were identified in MMR genes, including *MLH1* (n = 8), *MSH2* (n = 8), *MSH6* (n = 2), *PMS2* (n = 3), and *EPCAM* (n = 1) in twenty patients. The corresponding clinical and genomic details are shown in Table S2.

Mutations in non-LS genes are shown in Table S3. Pathogenic germline mutations of non-LS genes were *BRCA2* (n = 1), *BRIP1* (n = 1), *RAD50* (n = 1), and *MUTYH* (n = 1). One patient (YMCLynch016) had a mutation in both *MLH1* and *RAD50*. The patient was diagnosed with breast and endometrial cancer and had family history of gastric and small bowel cancer. Twenty-three VUS were identified in non-LS genes, including *CHEK2* (n = 5), *APC* (n = 4), *BRCA2* (n = 3), *RAD50* (n = 3), *BRIP1* (n = 2), *PALB2* (n = 2), *BARD1* (n = 1), *CDH1* (n = 1), *MUYTH* (n = 1), and *RAD51D* (n = 1), in 21 patients.

The performance of different clinical approaches to the prediction of LS is compared in Table S4. A combination of young age (<50 years) and family history of LS-associated cancers showed high sensitivity (0.93) but a low positive predictive value (0.20). The Amsterdam II criteria showed high specificity (0.94) and a high negative predictive value (0.92). Overall, the accuracy was higher for the Amsterdam II criteria than either MMR deficiency or MSI testing.

## 4. Discussion

This retrospective study investigated the prevalence and implications of germline mutations in patients with endometrial cancer who underwent multigene panel testing in Korea. In this cohort (n = 204), we identified 30 germline mutations consistent with LS. Excluding four close relatives who were in a proband’s family, the prevalence of LS was 13.0% (26/200) in this cohort (Table 2), which was higher than previously reported. In the nonselective population, the prevalence of LS ranged from 1.8–4.5% in the Western population [8,23] and from 1.2–4.4% in Korea [19,20]. In studies where patients were pre-screened based on various clinical factors, the incidence ranged from 4–9% [24,25]. The prevalence from our study is higher than the numbers reported in these previous studies.

Table 5 summarizes the prevalence of LS and non-LS cancers among endometrial cancer patients in recent studies that have used multigene panel tests similar to those used in this study [17,26,27]. In studies conducted in Western countries, the prevalence of LS has been reported to be 3.0–9.4%. There are several possible reasons for the relatively high prevalence of LS in this study compared to previous studies: (i) differences in the test methods, (ii) differences in the characteristics of the study populations, and (iii) differences in the application of “universal screening” using IHC and MSI tests.

The prevalence of different MMR gene mutations is known to vary in endometrial cancers. It is well known that the mutation rates of MMR genes are as follows: *MSH2*, 50–66%; *MLH1*, 24–40%; *MSH6*, 10–13%; and *PMS2*, <5% [28,29]. However, the prevalence of mutations in *MSH6* and *PMS2* in endometrial cancer patients with LS has been reported to be 52–72% in recent studies, as determined using multigene panel tests (Table 5) [17,26,27]. This is consistent with the prevalence of 40% observed in this study. Moreover, based on the present study, we found that patients with *MSH6* or *PMS2* mutations, compared to those with *MLH1* or *MSH2* mutations, were more likely to present with non-endometrioid histology (41.7% vs. 5.6%), which included clinically aggressive endometrial cancers, such as serous, neuroendocrine, clear cell, and undifferentiated cancers, which have a high risk of recurrence and mortality (Table 4). According to one study that reported histological data for LS patients, the frequency of non-endometroid histology is 0% (0/8) in patients with *MLH1* or *MSH2* mutations and 14.3% (3/21) in those with *MSH6* or *PMS2* mutations [27]. Further research is required on this topic.

One of the benefits of multigene panel testing is that it can help clinicians identify various genes with interesting clinical implications. For example, the presence of a PTEN mutation, especially when mutated concurrently with a PIK3CA and KRAS mutation, may predict sporadic MMR deficient endometrial cancer; the wild-type status of PTEN may be associated with LS-associated endometrial cancer [30,31]. As genetic sequencing is increasingly performed in recent studies, evidence on the prognostic and predictive role of genes or somatic interactions will continue to accumulate. With respect to mutational frequency, CHEK2 was reported as the most commonly mutated gene based on previous studies (Table 5). However, *CHEK2* mutations were not identified in the present study. Among LS-associated genes, the most frequent variant within LS genes in this Korean cohort was c.1758dupC (33.3%, 4/12) in the *MLH1* gene (Table 2). This mutation was also found at a high frequency in a previous Korean study (37.5%, 3/8) [18], and it has the potential to be a founder mutation in Korean patients with endometrial cancer associated with LS.

When we assessed the predictive performance of different approaches within our selected population of patients with endometrial cancer, the accuracy was higher for the Amsterdam II criteria than that for either MMR or MSI testing, despite the limited number of patients who underwent IHC/MSI testing (Table S4). These metrics highlight the importance of taking a thorough history of patients with endometrial cancer, even in the setting of universal screening of IHC markers in patients with LS. However, not all centers in Korea have adopted universal screening using the tumor IHC/MSI test due to insurance issues. Although MSI testing has the potential to be integrated into NGS testing in the future, at the current developmental stage, several technical difficulties are still associated with MSI testing [16]. Thus, an up-front NGS test may be more cost-effective than a step-by-step universal screening test. Moreover, even the Amsterdam II criteria are very stringent and may miss as many as 68% of patients with LS [15]. Furthermore, endometrial cancer is likely to be the first cancer diagnosed for approximately half of patients with LS [7]. Therefore, making an effort to identify patients with LS among those diagnosed with endometrial cancer may be a starting point for the early diagnosis of LS and the improved management of LS-associated cancer for patients and their families.

In summary, recent studies have shown that the prevalence of LS in endometrial cancer ranges from 3.0 to 9.4% (13.0% in this study); however, LS cases may be missed even with the application of universal screening. The proportion of *MSH6/PMS2*, where the non-endometrioid type with poor prognosis is found more frequently, appears to be high in endometrial cancer LS. Moreover, non-LS mutations are found in 2.0–7.1% of endometrial cancer patients. With recent developments in testing techniques, various genetic tests can now be provided at a low cost. Therefore, when endometrial cancer is diagnosed, establishing the pedigree and performing preemptive multigene panel testing using NGS may be suggested in the future as a strategy to identify patients with hereditary endometrial cancer including LS.

There are some limitations to the present study, including the retrospective study design. We enrolled all patients with endometrial cancer who underwent germline genetic testing; among all cases of newly diagnosed endometrial cancer patients across the three institutions over the 7-year study period, an average of 10.8% of endometrial cancer patients underwent germline NGS testing. Patients underwent genetic testing based on IHC results or a positive family history of LS-associated cancer, but we did not have a pre-specified protocol standardized across the different institutions. Thus, selection bias is likely to be present: the LS prevalence data may be exaggerated, and the data needs to be interpreted with caution with the specific study design in mind. A future prospective study where all endometrial cancer patients receive germline testing will help address the true prevalence of germline LS among endometrial cancer patients. Moreover, tumor testing, including IHC for MMR proteins and MSI tests, was performed for only a subset of patients. Moreover, there was no central pathological review and no *MLH1* methylation test results.

## 5. Conclusions

We present a comprehensive analysis of clinical IHC and genetic information from 204 patients with endometrial cancer. Based on a multigene panel test, we identified 30 patients with pathogenic mutations in LS genes and 22 patients with unclassified variants in LS genes. After excluding four relatives of a proband, the prevalence of LS was 13.0%. An analysis of clinical information showed that non-endometrial histology was more prevalent in patients with *MSH6* or *PMS2* mutations as compared to those with *MLH1* or *MSH2* mutations. Our findings also highlighted the importance of thorough history taking of patients with endometrial cancer, even in the setting of universal screening of IHC markers in patients with LS. To the best of our knowledge, this is the first retrospective study of multigene panel testing in patients with endometrial cancer in Korea, and our findings support the use of gene panel testing for endometrial cancer patients.

## Figures and Tables

**Table 1:** Clinico-pathological demographics of the endometrial cancer patients (n = 204).

Variables	n (%)
Age (years), median (range)	52 (23–84)
Histology	
Endometrioid	161 (78.9%)
Serous	17 (8.3%)
Clear cell	3 (1.5%)
Mixed	6 (2.9%)
Neuroendocrine	2 (1.0%)
Mesonephric	1 (0.5%)
Dedifferentiated	5 (2.5%)
Carcinosarcoma	5 (2.5%)
Sarcoma	4 (2.0%)
Tumor location	
Uterine corpus	172 (84.3%)
Lower uterine segment	32 (15.7%)
FIGO stage	
I	140 (68.6%)
II	14 (6.9%)
III	39 (19.1%)
IV	11 (5.4%)
Other LS-associated cancers *	
Yes	84 (41.2%)
No	120 (58.8%)
Family history ** of LS-associated cancers	
Yes	114 (55.9%)
No	90 (44.1%)
Met the Amsterdam II criteria	
Yes	26 (12.7%)
No	178 (87.3%)
Immunohistochemistry of MLH1/MSH2/MSH6/PMS2	
Tested	133
Loss of expression of at least one	57 (42.9%)
Intact expression of all four	76 (57.1%)
Microsatellite instability test	
Tested	96
Microsatellite instability-high	22 (22.9%)
Microsatellite stable or microsatellite instability-low	74 (77.1%)

FIGO, International Federation of Gynecology and Obstetrics; LS, Lynch syndrome. * Lynch syndrome-associated cancers: colorectal, ovarian, stomach, pancreatobiliary, urinary tract, small bowel, and brain (usually glioblastoma) cancer; sebaceous carcinoma, keratoacanthoma. ** Family history of Lynch syndrome-associated cancers within second-degree relatives.

**Table 2:** Detailed information of MMR gene germline pathogenic or likely pathogenic variants in endometrial cancer (n = 30).

Case	Age	Gene	Mutation	Histology	Tumor Location	Family History of Cancers	Amsterdam II Criteria
YMCLynch009	46	*MLH1*	c.1489dup	Endometrioid	UC	C (mother, sister)	Yes
YMCLynch006 *	39	*MLH1*	c.1758dup	Endometrioid	LUS	EM (sister), C (uncle, nephew), S (mother, uncle)	Yes
YMCLynch011 *	41	*MLH1*	c.1758dup	Endometrioid	UC	EM (sister), C (uncle, nephew), S (mother, uncle)	Yes
YMCLynch021	56	*MLH1*	c.1758dup	Endometrioid	UC	C (mother), GB (brother)	Yes
YMCLynch104	53	*MLH1*	c.1758dup	Endometrioid	UC	P (mother), C (brother, aunt)	Yes
CHALynch002	53	*MLH1*	c.1878_1881del	Endometrioid	UC	EM (sister), GB (sister)	No
YMCLynch008	33	*MLH1*	c.2206G > T	Endometrioid	UC	C (father, brother)	Yes
YMCLynch016	53	*MLH1*	c.306 + 1G > A	Endometrioid	UC	S (mother), Sm (brother)	Yes
YMCLynch005	44	*MLH1*	c.440_441insT	Serous	UC	C (sister), GB (mother), S (father)	Yes
YMCLynch028	40	*MLH1*	c.493del	Endometrioid	UC	C (father, grandfather)	Yes
YMCLynch020	38	*MLH1*	c.1036C > T	Endometrioid	UC	None	No
YMCLynch034	34	*MLH1*	c.790 + 1G > A	Endometrioid	LUS	C (father, aunt)	Yes
YMCLynch022	65	*MSH2*	c.1276 + 1G > A	Endometrioid	UC	C (brother)	No
SSCHLynch031	53	*MSH2*	c.187delG	Endometrioid	UC	None	No
YMCLynch024	48	*MSH2*	c.792 + 1del	Endometrioid	UC	Es (father)	No
YMCLynch017	49	*MSH2*	c.942 + 3A > T	Endometrioid	LUS	C (father, brother), S (uncle)	Yes
YMCLynch003	43	*MSH2*	c.942 + 3A > T	Endometrioid	UC	C (father)	No
CHALynch006	48	*MSH2*	c.1705_1706delGA	Endometrioid	UC	S (brother1, brother 2), H (father)	Yes
CHALynch008	38	*MSH6*	c.3261dupC	Endometrioid	UC	None	No
YMCLynch018	71	*MSH6*	c.3226C > T	Endometrioid	UC	C (brother), RC (brother)	No
***CHALynch011 #***	53	*MSH6*	c.3261dupC	Neuroendocrine	UC	C (sister, brother, sister 2), S (brother 1, brother 2, sister)	Yes
***CHALynch003 #***	61	*MSH6*	c.3261dupC	Serous	UC	C (sister, brother, sister 2), S (brother 1, brother 2, sister)	Yes
CHALynch012 **	56	*MSH6*	c.3539C > G	Dedifferentiated	UC	C (father), EM (daughter)	Yes
***YMCLynch027 ##***	37	*MSH6*	c.1295T > C	Endometrioid	UC	Ov (mother), EM (sister)	Yes
***YMCLynch012 ##***	45	*MSH6*	c.1295T > C	Endometrioid	UC	Ov (mother), EM (sister)	Yes
YMCLynch015	70	*MSH6*	c.3489dup	Endometrioid	LUS	EM (sister), C (brother), Th (son)	No
YMCLynch030	48	*MSH6*	c.3844del	Endometrioid	LUS	C (mother), Es (father), S (uncle 1), L (uncle 2)	Yes
CHALynch013 **	30	*MSH6*	c.3539C > G	Mixed	UC	C (grandfather), EM (mother)	Yes
CHALynch015	54	*PMS2*	c.1738A > T	Endometrioid	UC	None	No
YMCLynch031	38	*PMS2*	c.2444C > T	Mixed	UC	C (uncle), H (father)	Yes

MMR, mismatch repair; UC, uterine corpus; LUS, lower uterine segment; C, colorectal cancer; EM endometrial cancer; S, stomach cancer; GB, gall bladder cancer; P, pancreatic cancer; Sm, small bowel cancer; Es, esophageal cancer; H, hepatic cancer; RC, renal cancer; Ov, ovarian cancer; Th, thyroid cancer; L, lung cancer. *, **, #, ## bold italic cases indicate a Lynch syndrome family revealed by the proband.

**Table 3:** Comparison of patients with Lynch Syndrome and sporadic endometrial cancer.

Variables	Sporadic Cancer (n = 174)	Lynch Syndrome	*p*-Value
Age, median (range)	53 (23–84)	48 (30–71)	0.045
Histology			0.771
Endometrioid	137 (78.7%)	24 (80.0%)	
Serous	15 (8.6%)	2 (6.7%)	
Clear cell	3 (1.7%)	0	
Mixed	4 (2.3%)	2 (6.7%)	
Neuroendocrine	1 (0.6%)	1 (3.3%)	
Mesonephric	1 (0.6%)	0	
Dedifferentiated	4 (2.3%)	1 (3.3%)	
Carcinosarcoma	5 (2.9%)	0	
Sarcoma	4 (2.3%)	0	
Tumor location			0.586
Uterine corpus	148 (85.1%)	25 (83.3%)	
Lower uterine segment	26 (14.9%)	5 (16.7%)	
FIGO stage			0.357
I	121 (69.5%)	19 (63.3%)	
II	13 (7.5%)	1 (3.3%)	
III	30 (17.2%)	9 (30.0%)	
IV	10 (5.7%)	1 (3.3%)	
Other LS-associated cancers *			0.389
Yes	69 (39.7%)	15 (50%)	
No	105 (60.3%)	15 (50.0%)	
Family history ** of LS-associated cancers			0.001
Yes	88 (49.4%)	26 (86.7%)	
No	86 (50.6%)	4 (13.3%)	
Met the Amsterdam II criteria			<0.001
Yes	6 (3.5%)	20 (66.7%)	
No	168 (96.5%)	10 (33.3%)	

* Lynch syndrome-associated cancers: colorectal, ovarian, stomach, pancreatobiliary, urinary tract, small bowel, and brain (usually glioblastoma) cancer; sebaceous carcinoma; and keratoacanthoma. ** Family history of Lynch syndrome-associated cancers within second-degree relatives.

**Table 4:** Clinical comparison of patients with Lynch syndrome by high versus low-penetrance genes (n = 30).

Variables	High-Penetrance Genes (MLH1/MSH2, n = 18)	Low-Penetrance Genes (*MSH6*/*PMS2*, n = 12)	*p*-Value
Age (median, range)	47 (33–65)	40 (30–71)	0.407
Histology			0.026
Endometrioid	17 (94.4%)	7 (58.3%)	
Non-endometrioid	1 (5.6%)	5 (41.7%)	
Tumor location			0.660
Uterine corpus	15 (83.3%)	10 (83.3%)	
Lower uterine segment	3 (16.7%)	2 (16.7%)	
FIGO stage			0.694
I/II	11 (61.1%)	9 (75.0%)	
III/IV	7 (38.9%)	3 (25.0%)	
Other LS-associated cancers			0.264
Yes	11 (61.1%)	4 (33.3%)	
No	7 (38.9%)	8 (66.7%)	
Family history of LS-associated cancers			1
Yes	16 (88.9%)	10 (83.3%)	
No	2 (11.1%)	2 (16.7%)	
Amsterdam II criteria			1
Yes	12 (66.7%)	8 (66.7%)	
No	6 (33.3%)	4 (33.3%)	

**Table 5:** Literature review of multigene panel testing in patients with endometrial cancer.

	Ring et al. [26]	Levine et al. [27]	Karpel et al. [17]	Present Study
N	381	961	224	200 #
Country	USA	USA	USA	Korea
Year of publication	2016	2021	2022	2022
Number of genes tested	25	47	N/A	22
Age, median	61 *	62	57	52
Asian ethnicity	14 (3.7%)	9 (0.9%)	25 (11.2%)	200 (100%)
Endometrioid histology	289 (75.9%)	819 (85.2%)	148 (66.1%)	161 (80.5%)
Lynch syndrome genes	22 (5.8%)	29 (3.0)%	21 (9.4%)	26 (13.0%)
*MLH1*	3 (13.6%)	2 (6.9%)	4 (19.0%)	11 (42.3%)
*MSH2*	5 (22.7%)	6 (20.7%)	5 (23.8%)	6 (23.1%)
*MSH6*	6 (27.3%)	10 (34.5%)	7 (33.3%)	7 (26.9%)
*PMS2*	6 (27.3%)	11 (37.9%)	4 (19.0%)	2 (7.7%)
*EPCAM*	2 (9.1%)	29 (3.0)%	1 (4.8%)	
Non-Lynch syndrome genes	13 (3.4%)	68 (7.1%) **	13 (5.8%)	4 (2.0%)
*APC*	1 (7.7%)		2 (15.4%)	
*ATM*	1 (7.3%)	2 (2.9%)	2 (15.4%)	
*BARD1*	1 (7.3%)			
*BRCA1*	1 (7.3%)	4 (5.9%)	1 (7.7%)	
*BRCA2*	1 (7.3%)	6 (8.8%)	2 (15.4%)	1 (25.0%)
*BRIP1*	1 (7.3%)	6 (8.8%)		1 (25.0%)
*CHEK2*	4 (30.8%)	17 (25.0%)	6 (46.2%)	
*MUTYH*		15 (22.1%)		1 (25.0%)
*NBN*	1 (7.3%)	4 (5.9%)		
*PTEN*	1 (7.3%)			
*RAD50*				1 (25.0%)
*RAD51C*	1 (7.3%)	1 (1.5%)	1 (7.7%)	

* mean. # excluded four close relatives (sisters or daughters) in the proband’s family. ** other genes: *CDKN2A*, *SDHA*, *PALB2*, *NF1*, *MSH3*, and *NTHL1.*

## Data Availability

Due to privacy and ethical concerns, the data that support the findings of this study are available on request from the corresponding author.

## Supplementary Materials

The following supporting information can be downloaded at: https://www.mdpi.com/article/10.3390/cancers14143406/s1. Table S1: List of 22 genes; Table S2: Detailed information of MMR variants of unknown significance in endometrial cancer patients (n = 20); Table S3: Detailed information of pathogenic and likely pathogenic variants and variants of unknown significance in non-Lynch syndrome genes; Table S4: Comparison of the performance of the Amsterdam II criteria, immunohistochemistry-based assays, and microsatellite instability tests at predicting Lynch syndrome.
