# Supplementary material for: Identification of Lynch Syndrome in Patients with Endometrial Cancer Based on a Germline Next Generation Sequencing Multigene Panel Test
Source: Cancers (Basel). 2022 Jul 13;14(14):3406. doi: 10.3390/cancers14143406 (PMC9316192; doi:10.3390/cancers14143406)
Supplement: Supplementary file 1 [file cancers-14-03406-s001.zip › cancers-1792460-supplementary.pdf]

Table S1. List of 22 genes

APC
ATM
BARD1
BRCA1
BRCA2
BRIP1
CDH1
CHEK2
EPCAM
MLH1
MSH2
MSH6
MUTYH
NBN
PALB2
PMS2
RAD50
RAD51C
RAD51D
SMAD4
STK11
TP53

Table S2. Detailed information of MMR variants of unknown significance in endometrial cancer patients (n = 20)

Case	Age	Gene	Mutation	Histology	Tumor location	Family history of cancers	Amsterdam II criteria
YMCLynch013	58	*MLH1*	c.1153C>T	Endometrioid	UC	C (mother)	No
SSCHLynch015	63	*MLH1*	c.1153C>T	Endometrioid	UC	None	No
YMCLynch025	42	*MLH1*	c.413C>G	Mixed	LUS	P (father), S (aunt)	No
YMCLynch007	63	*MLH1*	c.5C>T	Endometrioid	UC	None	No
CHALynch005	42	*MLH1*	c.666T>G	Endometrioid	UC	UB (father)	No
SSCHLynch009	60	*MLH1*	c.2110G>C	Endometrioid	UC	None	No
SSCHLynch033	51	*MLH1*	c.2110G>C	Endometrioid	UC	S (father)	No
YMCLynch100	52	*MLH1*	c.649C>T	Endometrioid	UC	None	No
SSCHLynch025	47	*MSH2*	c.973_993del	Endometrioid	UC	None	No
CHALynch008	38	*MSH2*	c.14C>A	Endometrioid	UC	None	No
CHALynch009	63	*MSH2*	c.14C>A	Endometrioid	UC	None	No
YMCLynch010*	42	*MSH2*	c.599T>A	Endometrioid	UC	EM (sister 1), S (sister 1), C (sister 2)	No
YMCLynch010*	42	*MSH2*	c.1432C>G	Endometrioid	UC	EM (sister 1), S(sister 1), C (sister 2)	No
CHALynch010	52	*MSH2*	c.2509C>T	Endometrioid	UC	S (aunt)	No
CHALynch004	51	*MSH2*	c.2184_2186dup	Dedifferentiated	UC	C (mother, sister 1, sister 2, sister 3)	Yes
YMCLynch019	47	*MSH2*	c.599T>A	Endometrioid	UC	C (sister, twin), EM (sister)	No
SSCHLynch017	49	*MSH6*	c.3439-3C>A	Endometrioid	UC	None	No
CHALynch014	43	*MSH6*	c.3984G>T	Endometrioid	UC	None	No
CHALynch016	55	*PMS2*	c.1766A>G	Endometrioid	LUS	None	No
CHALynch017	54	*PMS2*	c.779C>A	Endometrioid	UC	None	No
CHALynch018#	38	*PMS2*	c.962T>C	Endometrioid	UC	S (grandfather)	No
CHALynch018#	38	*EPCAM*	c.298G>A	Endometrioid	UC	S (grandfather)	No

UC, uterine corpus; LUS, lower uterine segment; C, colorectal cancer; P, pancreatic cancer; S, stomach cancer; UB, urinary bladder cancer; EM endometrial cancer
*, # : had two variants of unknown significance

Table S3. Detailed information of pathogenic and likely pathogenic variants and variants of unknown significance in non-Lynch-syndrome genes

Case	Age	Histology	Gene	Variant	Pathogenicity	MMR gene	Variant	Pathogenicity	Family history of cancers	Other primary cancer
YMCLynch096	57	Serous	*BRCA2*	c.5576_5579del	Pathogenic					B, Ov
CHALynch007	47	Endometrioid	*BRIP1*	c.2702_2706del	LP				L (father), C (cousin)	
YMCLynch016	53	Endometrioid	*RAD50*	c.3277C>T	Pathogenic	*MLH1*	c.306+1A>G	Pathogenic	S (mother), Sm (brother)	B
SSCHLynch0011	44	Endometrioid	*MUTYH*	c.55C>T	Pathogenic					
CHALynch010*	52	Endometrioid	*APC*	c.757G>A	VUS	*MSH2*	c.2509C>T	VUS	S (aunt)	
CHALynch018	38	Endometrioid	*APC*	c.5257G>C	VUS	*PMS2*	c.962T>C	VUS	S (grandfather)	Ov
CHALynch005	42	Mixed	*APC*	c.5257G>C	VUS	*MLH1*	c.666T>G	VUS	UB (father)	
SSCHLynch007	69	Endometrioid	*APC*	c.7150T>A	VUS				Cx (dauther)	
SSCHLynch004	56	Endometrioid	*BARD1*	c.1479A>C	VUS				H (father)	
YMCLynch068	53	Sarcoma	*BRCA2*	c.946A>C	VUS					
CHALynch057	57	Endometrioid	*BRCA2*	c.7051G>A	VUS				Ut (mother)	B, P
CHALynch058	58	Carcinosarcoma	*BRCA2*	c.7706G>A	VUS					
CHALynch010*	52	Endometrioid	*BRIP1*	c.1442G>A##	VUS				S (aunt)	
CHALynch046	59	Endometrioid	*BRIP1*	c.2440C>T	VUS				L ((brother)	
CHALynch014#	43	Endometrioid	*CDH1*	c.1514T>C	VUS	*MSH6*	c.3984G>T	VUS		Ov
CHALynch043	63	Endometrioid	*CHEK2*	c.4T>A	VUS				S (father, sister, brother 1)	
YMCLynch025	42	Mixed	*CHEK2*	c.8G>A	VUS	*MLH1*	c.413C>G	VUS	Pr (father), S (aunt)	
CHALynch045	47	Neuroendocrine	*CHEK2*	c.1009-7T>G	VUS					
YMCLynch055	27	Endometrioid	*CHEK2*	c.1111C>T	VUS				L (mother)	Ov
CHALynch026	70	Endometrioid	*CHEK2*	c.1561C>T	VUS					
CHALynch049	50	Endometrioid	*MUTYH*	c.934-2A>G	VUS				S (father)	
CHALynch023	36	Endometrioid	*PALB2*	c.377A>G	VUS				Ut (aunt)	Th
YMCLynch059	50	Endometrioid	*PALB2*	c.2791C>T	VUS				B (mother), Th (sister)	B
CHALynch009	63	Endometrioid	*RAD50*	c.353T>C	VUS	*MSH2*	c.14C>A	VUS		
CHALynch021	59	Endometrioid	*RAD50*	c.1211A>G	VUS				S (uncle)	
CHALynch024	60	Endometrioid	*RAD50*	c.3790C>T	VUS					C
CHALynch014#	43	Endometrioid	*RAD51D*	c.932T>A	VUS					

LP, likely pathogenic; VUS, variants of unknown significance; B, breast cancer; Ov, ovary cancer; L, lung cancer; C, colorectal cancer; S, stomach cancer; Sm, small bowel cancer; VUS, variant of unknown significance; UB, urinary bladder tumor; Cx, cervix cancer; H, hepatic cancer; Ut, uterine cancer; P, pancreatic cancer; Pr, prostate cancer; Th, thyroid cancer.

*, # : had two variants of unknown significance

this variant could be regarded as likely benign in the Asian population.

Table S4. Comparison of the performance of the Amsterdam II criteria, immunohistochemistry-based assays, and microsatellite instability tests at predicting Lynch syndrome

		Sporadic endometrial cancer	Endometrial cancer with germline LS	Performance metrics
Demographics: Age <50 with positive family history of cancer	No	61	2	Accuracy: 0.436<br>Sensitivity: 0.933<br>Specificity: 0.351<br>PPV: 0.199<br>NPV: 0.968
	Yes	113	28	
Satisfies Amsterdam II criteria	No	164	14	Accuracy: 0.882<br>Sensitivity: 0.533<br>Specificity: 0.943<br>PPV: 0.615<br>NPV: 0.921
	Yes	10	16	
MMR status	Proficient	73	3	Accuracy: 0.662<br>Sensitivity: 0.833<br>Specificity: 0.635<br>PPV: 0.263<br>NPV: 0.961
	Deficient	42	15	
MSI status	Low	70	4	Accuracy: 0.813<br>Sensitivity: 0.667<br>Specificity: 0.833<br>PPV: 0.364<br>NPV: 0.946
	High	14	8	

MMRd, results of immunohistochemistry analysis of MLH1, MSH2, MSH6, and PMS2; MSI, results of microsatellite instability test; PPV, positive predictive value; NPV, negative predictive value.
